# The Role of the Intestinal Microbiome in Chronic Psychosocial Stress-Induced Pathologies in Male Mice

**DOI:** 10.3389/fnbeh.2018.00252

**Published:** 2018-10-26

**Authors:** Dominik Langgartner, Carolyn A. Vaihinger, Melanie Haffner-Luntzer, Julia F. Kunze, Anna-Lena J. Weiss, Sandra Foertsch, Stephanie Bergdolt, Anita Ignatius, Stefan O. Reber

**Affiliations:** ^1^Laboratory for Molecular Psychosomatics, Clinic for Psychosomatic Medicine and Psychotherapy, University of Ulm, Ulm, Germany; ^2^Institute of Orthopedic Research and Biomechanics, University Medical Center Ulm, Ulm, Germany

**Keywords:** chronic psychosocial stress, chronic subordinate colony housing (CSC), anxiety, bone homeostasis, inflammation, microbiome, fecal transplantation

## Abstract

Chronic psychosocial stress is a risk factor for the development of physical and mental disorders accompanied or driven by an activated immune system. Given that chronic stress-induced systemic immune activation is lacking in germ-free and antibiotics-treated mice, a causal role of the gut microbiome in the development of stress-related disorders is likely. To address this hypothesis in the current study we employed the chronic subordinate colony housing (CSC, 19 days) paradigm, a pre-clinically validated mouse model for chronic psychosocial stress, known to alter the gut microbial signature and to induce systemic low-grade inflammation, as well as physical and mental abnormalities. In detail, we investigated if (i) CSC-induced alterations can be prevented by repeated transplantation of feces (FT) from non-stressed single-housed control (SHC) mice during CSC exposure, and (ii) if the transplantation of a “stressed” CSC microbiome is able to induce CSC effects in SHC mice. Therefore, we repeatedly infused SHC and CSC recipient mice rectally with SHC donor feces at days 4 and 11 of the CSC paradigm and assessed anxiety-related behavior on day 19 as well as physiological, immunological, and bone parameters on day 20. Furthermore, SHC and CSC recipient mice were infused with CSC donor feces at respective days. To exclude effects of rectal infusions *per se*, another set of SHC and CSC mice was infused with saline, respectively. Our results showed that transplantation of SHC feces had mild stress-protective effects, indicated by an amelioration of CSC-induced thymus atrophy, anxiety, systemic low-grade inflammation, and alterations in bone homeostasis. Moreover, transplantation of CSC feces slightly aggravated CSC-induced systemic low-grade inflammation and alterations in bone homeostasis in SHC and/or CSC animals. In conclusion, our data provide evidence for a role of the host’s microbiome in many, but not all, adverse consequences of chronic psychosocial stress. Moreover, our data are consistent with the hypothesis that transplantation of healthy feces might be a useful tool to prevent/treat different adverse outcomes of chronic stress. Finally, our data suggests that stress effects can be transferred to a certain extend via FT, proposing therapeutic approaches using FT to carefully screen fecal donors for their stress/trauma history.

## Introduction

Chronic psychosocial stress is an acknowledged risk factor for the development of various somatic and affective disorders, including inflammatory bowel disease (IBD) and posttraumatic stress disorder (PTSD) (Duffy et al., 1991; Agid et al., 1999; Bitton et al., 2003; de Kloet et al., 2005; Bernstein et al., 2010; Virtanen and Kivimaki, 2012; Virtanen et al., 2012; Langgartner et al., 2015; Reber et al., 2016a,b). However, although chronic stress results in a dysregulation of almost all neuro-immuno-endocrine systems of an organism, the exact mechanisms underlying these stress-related disorders are still largely unknown. A relatively novel, but promising, hypothesis in this context is that the pathogenic potential of chronic stress is, at least in part, mediated by the intestinal microbiome (Mackos et al., 2015, 2017; Tarr et al., 2015; Galley et al., 2017; Lafuse et al., 2017).

According to recent findings, the ratio of bacterial to eukaryotic cells in the human body is approximately 1:1 (Sender et al., 2016), resulting in an overall microbial mass comparable to the weight of the human brain (Stilling et al., 2014, 2016). The majority of these microorganisms resides in the gut (Sender et al., 2016), comprising around 150 times more genes than the human genome (Qin et al., 2010). Thus, it is not surprising that the gut microbiome is able to influence the host in a variety of parameters. For instance, rodent studies have shown that the transfer of donor microbiome strongly affects recipients’ behavior, physiology and metabolism (Bercik et al., 2011; Collins et al., 2013). Furthermore, fecal transplantation (FT) from depressed patients into rats pretreated with antibiotics induces depressive-like behavior (Kelly et al., 2016) and colonizing germ-free (GF) mice with the gut microbiota of IBD patients causes barrier dysfunction and innate immune activation (De Palma et al., 2017). Moreover, colonization of GF mice with a specific-pathogen free (SPF) microbiome promotes an increased bone formation and resorption (Yan et al., 2016).

In turn, factors like diet (Singh et al., 2017), age (Mariat et al., 2009), pharmaceutics (Langdon et al., 2016), but also stressor exposure are well-known to affect the gut microbiome, although the latter is very stable over time (Allison and Martiny, 2008). For instance, PTSD in humans (Hemmings et al., 2017) and psychological stressors in rodents (O’Mahony et al., 2009; Bailey et al., 2010, 2011; Cui et al., 2016) are associated with compositional changes of the gut microbiome, paralleled by immune activation as indexed by increased circulating interleukin (IL)-6 and monocyte chemoattractant protein (MCP)-1 (Bailey et al., 2011) levels. Of note, these bidirectional processes between the host and the microbiome are referred to as the “microbiome-gut-brain-axis” (MGBA) and involve different neuro-immuno-endocrine mediators, including the vagus nerve, stress hormones, the immune system as well as the tryptophan metabolism and microbial metabolites (Cryan and Dinan, 2012).

The “chronic subordinate colony housing” (CSC) paradigm is a well-characterized animal model for PTSD in male mice (Reber et al., 2016a) and causes chronic low-grade inflammation (Langgartner et al., 2018), disturbance of bone homeostasis (Foertsch et al., 2017), general and social anxiety (Slattery et al., 2012; Langgartner et al., 2017), and, in the presence of colonic pathobionts like *Helicobacter* spp. (Langgartner et al., 2017), spontaneous colitis (Reber et al., 2007, 2011, 2016b; Langgartner et al., 2017). Although CSC further causes an increase in β-diversity (microbial diversity between different samples) and a decrease in α-diversity (microbial diversity within one sample) within the gut microbiome (Reber et al., 2016a,b), as described for many chronic stressors (Bailey, 2014), it is not known whether these compositional changes are critically involved in the above described behavioral, physiological, and immunological consequences of CSC.

Therefore, we in the current study aimed to test in a first approach if repeated transfer of a healthy SPF gut microbiome from non-stressed single-housed control (SHC) mice into CSC mice using FT is able to prevent CSC-induced behavioral, physiological, and immunological consequences. In a second approach, we also tested if transplantation of a “stressed” CSC microbiome is able to induce well-known CSC effects in SHC mice.

## Materials and Methods

### Animals

Male C57BL/6N (weighing 19–22 g) mice were used as both fecal donor and fecal recipient animals and male CD-1 mice (weighing 30–35 g) were used as dominant residents. All animals were obtained from Charles River (Sulzfeld, Germany). After delivery, donor as well as recipient mice were individually housed in a SPF animal facility under a 12 h/12 h light dark cycle, 22°C, 60% humidity, and had free access to tap water and standard mouse diet. The animal study was carried out in accordance with the relevant guidelines and regulations and was approved by the Federal Animal Care and Use Committee [Regierungspraesidium Tuebingen, Germany (permit No.: 1195)].

### Experimental Procedures

One to two weeks after arrival, mice were either exposed to the CSC paradigm (days 1–20) or kept as SHC mice (days 1–20; for details, see section “Chronic Subordinate Colony Housing (CSC) Procedure”). All experimental mice were weighed on days -6, 1, 4, 8, 11, 15, 19, and 20. Three sets of CSC and SHC animals were used in this study. In detail, on days 4 and 11, animal set 1 (SHC: *n* = 20, CSC: *n* = 16) was infused rectally with saline, animal set 2 (SHC: *n* = 12, CSC: *n* = 12) with SHC donor feces, and animal set 3 (SHC: *n* = 12, CSC: *n* = 12) with CSC donor feces. All animals were tested for anxiety-related behavior in the open-field/novel object (OF/NO) test on day 19 of CSC between 07:00 and 10:00 AM, and sacrificed in the morning of day 20 between 07:00 and 10:00 AM following brief CO_2_ anesthesia. Afterward, absolute adrenal- and thymus weight, plasma corticosterone (CORT), CORT production from *in vitro* adrenocorticotropic hormone (ACTH)-stimulated adrenal explants, and the histological damage score were assessed in all these mice. In addition, local [mRNA expression of colonic tumor necrosis factor-α (TNFα), interferon-γ (IFNγ), cathelin-related antimicrobial peptide (CRAMP), colonic protein expression of F4/80 and cluster of differentiation molecule (CD11b)] and systemic [keratinocyte chemoattractant (KC), IL-6, and monocyte chemotactic protein-1 (MCP-1)] inflammatory parameters were measured in some mice of each animal set [local: Set 1 (SHC: *n* = 8, CSC: *n* = 4); Set 2 (SHC: *n* = 12, CSC: *n* = 12); Set 3 (SHC: *n* = 12, CSC: *n* = 12); systemic: Set 1 (SHC: *n* = 8, CSC: *n* = 4); Set 2 (SHC: *n* = 4, CSC: *n* = 4); Set 3 (SHC: *n* = 8, CSC: *n* = 4)]. Moreover, bone homeostasis [tibia length, femoral growth-plate width, trabecular thickness, trabecular bone mineral density (BMD), trabecular bone volume/tissue volume (BV/TV) and trabecular number] parameters were assessed in some mice of each animal set [Set 1 (SHC: *n* = 8, CSC: *n* = 4); Set 2 (SHC: *n* = 4, CSC: *n* = 4); Set 3 (SHC: *n* = 8, CSC: *n* = 8)]. Two animals (CSC-saline) died due to unknown reasons before day 19, and thus were excluded from all parameters. One animal (CSC-saline) died due to unknown reasons between d19 and d20, and thus was excluded from the physiological parameters. Two adrenal glands (CSC-saline) were injured during the preparation procedure and thus excluded from the analysis. Of note, donor mice were exposed to either SHC or CSC exposure, starting on the same day as SHC or CSC exposure of respective recipient mice. In other words, feces infused on days 4 and 11 into recipient mice originated from donor mice exposed to SHC or CSC for either 4 or 11 days, respectively. Donor feces were collected from overall *n* = 28 SHC and *n* = 24 CSC mice. For more details, see section “Preparation of Fecal Suspension and Transplantation Procedure.”

### Chronic Subordinate Colony Housing (CSC) Procedure

The CSC procedure was performed as described in previous publications (Reber et al., 2007; Langgartner et al., 2015). Briefly, experimental mice were weighed and assigned to either the SHC or the CSC group, matched according to their body weight. The bedding of SHC mice was changed once a week. In order to induce chronic psychosocial stress, a group of four CSC mice was housed together with a dominant male CD-1 mouse for 19 consecutive days. To avoid habituation, CSC mice were placed into the home cage of a novel dominant male CD-1 mouse on days 8 and 15. All mice were weighed twice a week and received a rectal infusion of either saline (animal set 1), SHC donor feces (animal set 2) or CSC donor feces (animal set 3) on days 4 and 11 of CSC, respectively.

### Preparation of Fecal Suspension and Transplantation Procedure

To collect feces from donor animals, mice of both the SHC and CSC donor group were housed individually (without bedding) during stool pellet collection (approximately 15–20 min; 2–3 stool pellets per mouse). Collected and pooled (per group) fecal pellets were stored on ice (4°C) until homogenization in isotonic saline solution (1 part feces + 4 parts saline; Fresenius Kabi, Bad Homburg, Germany) using the Vortex Genie 2 (Scientific Industries Inc., Bohemia, NY, United States). To prevent congesting of the feeding needle (Heidelberg, Germany), which was used to rectally infuse the fecal suspension, the homogenate was filtered through a cell strainer (70 μm, Corning, Durham, NC, United States) to remove coarse particles. Subsequently (between 08:00 and 10:00 AM), recipient mice were rectally infused with 100 μl of the respective solution [saline (animal set 1), SHC donor feces (animal set 2) or CSC donor feces (animal set 3)].

### Open-Field/Novel Object Test

To assess CSC effects on anxiety-related behavior, SHC and CSC animals were exposed to the OF/NO test on day 19 of CSC exposure. Briefly, the arena (45 cm length × 27 cm width × 27 cm height) was subdivided into an inner (27 cm × 9 cm) and an outer zone. The arena was cleaned thoroughly before each trial. Within each trial, the mouse was placed into the inner zone and was allowed to explore the arena for 5 min. After 5 min of open-field exploration, a plastic round object (diameter: 3.5 cm; height: 1.5 cm) was placed in the middle of the inner zone. The mouse now was allowed to explore the arena containing the unfamiliar object for 5 min. In the open-field test, the number of inner zone entries and the time spent in the corners of the arena (measurements of anxiety-related behavior) as well as the distance moved (measurement of general activity) were assessed. In the novel object test, the number of object explorations and the time spent in the corners of the arena as well as the distance moved (all parameters of anxiety-related behavior) were analyzed. All parameters were analyzed using EthoVision XT (Version 9, Noldus Information Technology, Wageningen, Netherlands). The test was performed between 07:00 and 10:00 AM under white light conditions (350 lux).

### Trunk Blood Sampling

All mice were euthanized by decapitation in the morning of day 20. In detail, within 3 min after removing the cage from the animal room, mice were rapidly euthanized by decapitation following brief CO_2_-exposure. Trunk blood was collected in ethylenediaminetetraacetic acid (EDTA)-coated tubes (Sarstedt, Nuembrecht, Germany) and stored on ice until centrifugation. Tubes were centrifuged at 4°C (5,000 × *g*, 10 min). Plasma samples were stored at -20°C until further analysis.

### Assessment of Adrenal- and Thymus Weight

Adrenal glands were removed, pruned of fat, weighed, and stored in ice-cold Dulbecco’s Modified Eagle Medium [DMEM/F-12, Life Technologies, Inc., Grand Island, NY, United States; supplemented with 0.1% bovine serum albumin (BSA; Biomol, Hamburg, Germany)], until used for *in vitro* ACTH stimulation. Thymus was removed and stored in ice-cold phosphate buffered saline (PBS; Life Technologies) until all animals were euthanized. Subsequently, thymus glands were pruned of fat and weighed separately.

### *In vitro* ACTH Stimulation of Adrenal Explants

Adrenal *in vitro* ACTH stimulation was performed as described previously (Uschold-Schmidt et al., 2012). Briefly, after pruning from fat and weighing, adrenals were stored in ice-cold DMEM (DMEM/F-12, Life Technologies, Inc.) supplemented with 0.1% BSA, until further treatment. Adrenal glands were then cut into two halves (with each containing a cortical and medullary part), weighed again and pre-incubated in 200 μl DMEM/F-12 for 4 h (37°C, 5% CO_2_). Afterward, culture medium was replaced by fresh medium, thereby each half of one adrenal gland was incubated with either medium containing saline (basal) or medium containing ACTH (100 nM; representing a pharmacological dose of ACTH) for 6 h (37°C, 5% CO_2_). After 6 h of stimulation, supernatants were carefully removed and stored at -20°C until further analysis. Samples were analyzed using a commercially available ELISA for CORT (analytical sensitivity: <0.564 ng/ml, intra-assay and inter-assay coefficients of variation ≤6.35%; IBL International, Hamburg, Germany). CORT concentrations were calculated in relation to the weight of the respective adrenal explant.

### Enzyme-Linked Immunosorbent Assay (ELISA) for Plasma CORT and Multiplex Cytokine Analysis

Plasma samples were analyzed using a commercially available ELISA for CORT (analytical sensitivity: <0.564 ng/ml, intra-assay and inter-assay coefficients of variation ≤6.35%; IBL International, Hamburg, Germany). Further, plasma concentrations of KC, IL-6 and MCP-1, were determined using a mouse multiplex cytokine kit (ProcartaPlex, eBioscience, Frankfurt, Germany). Data analysis was performed using the Luminex^®^ 100 Total System (Bio-Rad Laboratories, Hercules, CA, United States).

### Quantitative Real-Time PCR Analysis of TNFα, IFNγ, and CRAMP mRNA in Colonic Tissue

In order to determine CSC and/or FT effects on colonic TNFα, IFNγ, and CRAMP mRNA expression, one piece of colonic tissue was frozen at -80°C until starting the RNA isolation. RNA was isolated using the RNeasy Mini kit (Qiagen, Valencia, CA, United States) according to the manufacturer’s instructions, including DNase I (Qiagen, Valencia, CA, United States) digestion. The amount of RNA was measured using a Nanodrop 2000 (Thermo Fischer Scientific, Waltham, MA, United States). 1 μg of RNA was used to generate cDNA employing random primers (High-capacity cDNA reverse transcription kit, Applied Biosystems, Thermo Fischer Scientific, Waltham, MA, United States) and additional RNase inhibitor “RNase out” to reduce RNase activity (Invitrogen, Thermo Fischer Scientific, Waltham, MA, United States). Real Time PCR was performed using a ViiA7 Real-Time PCR System (Applied Biosystems, Thermo Fischer Scientific, Waltham, MA, United States) using Platinum SYBR Green (Invitrogen, Thermo Fisher Scientific, Waltham, MA, United States). The primers (Sigma-Aldrich, St. Louis, MO, United States) used were Ribosomal Proteins (RPL; Housekeeper) (forward: 5′ CCTGCTGCTCTCAAGGTT 3′; reverse: 5′ TGGCTGTCACTGCCTGGTACTT 3′), TNFα (forward: 5′AGGGGCCACCACGCTCTTCT3′, reverse: 5′TGAGTGTGAGGGTCTGGGCCAT3′), CRAMP (forward: 5′CAGCCCTTTCGGTTCAAGAA3′, reverse: 5′CCCACCTTTGCGGAGAAGT3′) and IFNγ (forward: 5′TGCTGATGGGAGGAGATGTCT3′, reverse 5′TGCTGTCTGGCCTGCTGTTA3′). For the quantification of results, the corresponding Applied Biosystems Software was used and ΔΔ Ct analysis was performed.

### Protein Analysis of F4/80 and CD11b in Colonic Tissue

To measure colonic protein expression of F4/80 and CD11b, one piece of colonic tissue was frozen at -80°C until further processing. Protein extraction was performed by homogenizing the tissue in ethylenediaminetetraacetic acid (EDTA) lysis buffer [50 mM EDTA, 250 mM NaCl, 0.5 mM 4-(2-hydroxyethyl)-1-piperazineethanesulfonic acid, 0.5% Igepal, 10% Complete Mini Protease Inhibitor (Roche Diagnostics GmbH, Mannheim, Germany)]. Total protein concentration was determined using a commercially available detection kit (Bicinchoninic Acid Protein Assay Kit, Thermo Scientific, Rockford, IL, United States). Western blot analysis was performed by loading 30 μg of protein per colonic sample onto sodium dodecyl sulfate polyacrylamide gels (10%) and subsequent transfer on nitrocellulose membranes (Amersham Protran Premium 0.45 μm NC, GE Healthcare Life science, Freiburg, Germany). Membranes were then blocked for 1 h at room temperature (RT) in 5% milk powder (Carl Roth GmbH + Co. KG, Karlsruhe, Germany) diluted in Tris-buffered saline (TBS) with 0.05% Tween-20 (TBST; VWR, Darmstadt, Germany), and afterward incubated with primary F4/80 antibody (1:1000, Santa Cruz Biotechnology, Dallas, TX, United States). overnight at 4°C. Horseradish peroxidase (HRP)-conjugated goat anti-rabbit antibody (1:2000, Cell Signaling Technology, New England Biolabs GmbH, Frankfurt am Main, Germany) was used as secondary antibody (1 h, RT). After incubation with ECL Western Blotting Detection Reagents (GE Healthcare, Freiburg, Germany), chemiluminescence was digitalized and analyzed using Molecular Imager^®^ ChemiDoc^TM^ XRS+ system and Image Lab^TM^ (Bio-Rad Laboratories GmbH, Munich, Germany), respectively. After that, membranes were stripped using Re-Blot Plus Strong Antibody Stripping Solution (Millipore GmbH, Schwalbach, Germany), blocked twice and incubated with primary β-Tubulin (1:1000, 1 h at RT; Cell Signaling Technology, Danvers, MA, United States). HRP-conjugated anti-rabbit antibody (1:1000) was again used as secondary antibody (30 min, RT). Following visualization and digitalization, membranes were again stripped and this time incubated with CD11b antibody (1:2000; Abcam^®^, Cambridge, United Kingdom) over night at 4°C. Following incubation with HRP-conjugated anti-rabbit antibody (1:2000), visualization and digitalization were again performed as described above. Bands were detected at 160 kDa (F4/80), 170 kDa (CD11b), 55 kDa β-Tubulin as described by the manufacturers. The expression of F4/80 and CD11b was normalized to the respective β-tubulin protein expression.

### Determination of the Histological Damage Score of the Colon

To assess CSC effects on the histological damage of the colon, the colon was removed after decapitation and cleaned. Histological damage score was assessed as described previously (Reber et al., 2007) with slight modifications. One centimeter of the distal part of the distal third of the colon was cut longitudinally and fixed in 5% formalin for 48 h. Fixed tissue was then embedded in paraffin and cut longitudinally. Two 3 μm hematoxylin–eosin stained sections taken at 100 μm distance were evaluated by histological scoring performed by an investigator blind to treatment. Each individual score represented the mean of the sections. Histological damage score ranges from 0 to 8 and represents the sum of the epithelium score (0: normal morphology; 1: loss of goblet cells; 2: loss of goblet cells in large areas; 3: loss of crypts; 4: loss of crypts in large areas) and infiltration score (0: no infiltration; 1: infiltrate around crypt bases; 2: infiltrate reaching to lamina muscularis mucosae; 3: extensive infiltration reaching the lamina muscularis mucosae and thickening of the mucosa with abundant edema; 4: infiltration of the lamina submucosa).

### Assessment of Bone Homeostasis

Both femurs and right tibia were removed for further analysis and stored in 4% formalin. Tibia lengths were assessed using a digital precision caliper. Left femurs were subjected to μCT analysis as described previously (Foertsch et al., 2017). Briefly, scanning was done at 50 kV and 200 mA, voxel resolution was set to 8 μm. Analysis was conducted according to ASBMR guidelines (Bouxsein et al., 2010). BMD was assessed using two phantoms with defined hydroxyapatite (HA) contents (250 and 750 mg/cm^3^). The threshold for mineralized tissue was set at 390 mg HA/cm^3^ for trabecular bone. Right femurs were subjected to decalcified histology as described previously (Haffner-Luntzer et al., 2014). Sections of 7 μm were stained with Safranin-O to analyze the width of the growth plate by Osteomeasure system (Osteometrix).

### Statistics

For statistical comparisons, the software package IBM SPSS statistics (version 22.0; IBM Corporation, Armonk, NY, United States) was used. Kolmogorov–Smirnov test using Lilliefors’ correction was employed to test normal distribution of all acquired data sets. Outliers in normally distributed data sets were identified using Grubbs’ test and excluded from further analysis (Grubbs, 1969). Outliers were identified in the following normally distributed datasets: absolute adrenal weight: Saline CSC (one animal), CSC recipient SHC (one animal); absolute thymus weight: saline CSC (one animal), CSC recipient SHC (one animal); distance moved NO: saline CSC (one animal). Normally distributed data sets were subsequently analyzed using parametric statistics, i.e., parametric Student’s *t*-test [one factor, two independent samples; readouts: Physiology (absolute thymus weight; set 1)]; two-way ANOVA [two factors, two or more independent samples; readouts: Physiology (absolute adrenal weight, absolute thymus weight); Behavior (distance moved OF, time in corners OF, entries to OF, distance moved NO)]. Non-normally distributed data sets were analyzed using non-parametric statistics, i.e., Mann–Whitney *U* test [one factor, two independent samples; readouts: Physiology (body weight gain, plasma CORT, *in vitro* adrenal CORT production); Behavior (Time in corners NO, NO exploration); Intestinal inflammation (Histological damage score, F4/80 protein expression, CD11b protein expression, relative TNFα mRNA, relative IFNγ mRNA, relative CRAMP mRNA); Systemic inflammation (Plasma KC, plasma Il-6, plasma MCP-1); Bone homeostasis (Tibia length, growth plate thickness, trabecular thickness, trabecular BMD, bone vol./tissue vol., trabecular number)]; Kruskal–Wallis *H*-test [KWH-test; one factor, more than two independent samples; readouts: Physiology (body weight gain, plasma CORT); Behavior (Time in corners NO, NO exploration); Intestinal inflammation (Histological damage score, F4/80 protein expression, CD11b protein expression, relative TNFα mRNA, relative IFNγ mRNA, relative CRAMP mRNA); Systemic inflammation (Plasma KC, plasma Il-6, plasma MCP-1); Bone homeostasis (Tibia length, growth plate thickness, trabecular thickness, trabecular BMD, bone vol./tissue vol., trabecular number)]. All tests comparing more than two samples were followed, when a significant main effect was found, by *post hoc* analysis using Bonferroni pairwise comparison. For graphical illustration, the software package SigmaPlot (version 13.0; Systat Software Inc., San Jose, CA, United States) was used. Normally distributed data are presented as bars (mean + SEM). Non-normally distributed data are presented as box plots. Solid line represents the median, dashed line represents the mean for each data set. Lower box indicates 25th_,_ upper box indicates 75th percentile, 10th (lower error bar) and 90th percentile (upper error bar) as well as possible outliers beyond the percentiles (indicated by closed circles) are also shown. The level of significance was set at *P* < 0.05.

## Results

### Infusion of SHC Donor Feces Slightly Ameliorates CSC-Induced Behavioral Effects

**FIGURE 1:**
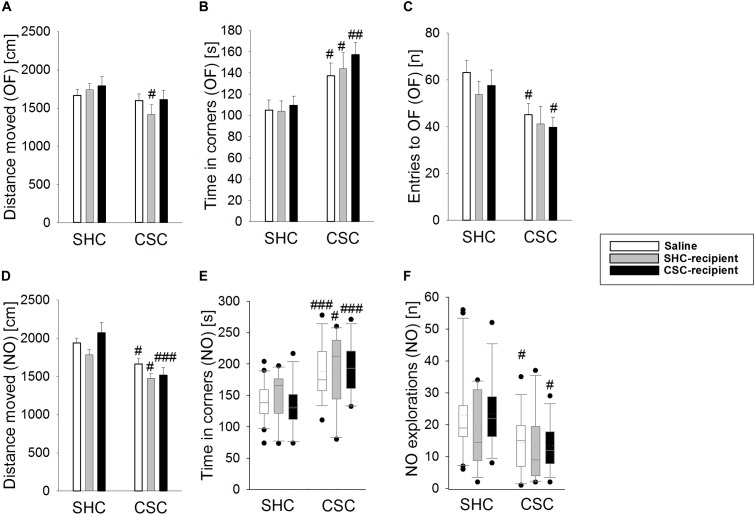
Effects of fecal transplantation (FT) on chronic subordinate colony (CSC) housing-induced anxiety-related behavior. Anxiety-related behavior was assessed in the Open Field/Novel Object (OF/NO) test in the morning of day 19. Single-housed control (SHC)-infused CSC compared with respective SHC animals showed a decreased distance moved during the OF test **(A)**. Compared to the respective SHC groups, all CSC groups spent significantly more time in the corners during OF exposure **(B)**. In contrast to saline- and CSC-infused CSC mice, SHC-infused CSC did not show a decreased number of entries into the OF **(C)**. Compared to the respective SHC groups, all CSC groups showed a decreased locomotion **(D)** and an increased time in the corners during NO exposure **(E)** in the NO test. However, in contrast to saline- and CSC-infused CSC mice, SHC-infused CSC animals did not show a decreased number of NO explorations **(F)** during the NO test, when compared to their respective SHC group. SHC (*n* = 12–20); CSC (*n* = 12–14). Parametric data are presented as mean + SEM. Non-parametric data are represented as box-plots. Solid line represents the median, dotted line represents the mean for each data set. Lower boxes indicate 25th, upper boxes indicate 75th percentile, 10th (lower error bar), and 90th percentile (upper error bar) as well as possible outliers beyond the percentiles (indicated by closed circles) are also shown. ^#^*P* ≤ 0.05, ^##^*P* ≤ 0.01, ^###^*P* ≤ 0.001 versus respective SHC.

Chronic subordinate colony housing increased anxiety-related behavior in the OF/NO test performed on day 19 of CSC (Figures 1A–F) in all groups compared to the respective SHC group, indicated by a significant main effect of the factor stress in the time spent in the corners during the OF test (two-way ANOVA; main effect for stress: *F*_1,76_ = 18.207; *P* < 0.001; Bonferroni: saline-infused: *P* = 0.03; SHC-infused: *P* = 0.021; CSC-infused: *P* = 0.006; Figure 1B) as well as the distance moved during the NO test (two-way ANOVA; main effect for stress: *F*_1,75_ = 28.876; *P* < 0.001; Bonferroni: saline-infused: *P* = 0.016; SHC-infused: *P* = 0.018; CSC-infused: *P* < 0.001; Figure 1D). Moreover, all CSC vs. SHC groups spent more time in the corners of the arena during the NO-test [Saline-treated: *P* = 0.001 (MWU); SHC-recipient: *P* = 0.039 (MWU); CSC-recipient: *P* = 0.001 (MWU); Figure 1E].

The number of entries to the open field (two-way ANOVA; main effect of stress: *F*_1,76_ = 11.169; *P* = 0.001; Figure 1C) was only significantly decreased in saline-treated CSC vs. SHC (Bonferroni: *P* = 0.018) as well as CSC-recipient CSC vs. SHC animals (Bonferroni: *P* = 0.045). Moreover, saline-infused CSC (MWU-test: *P* = 0.027) and CSC-recipient CSC (MWU-test: *P* = 0.012) but not SHC-recipient CSC animals displayed a reduced number of novel object explorations during the NO test (Figure 1F) when compared to their respective control group. However, SHC-recipient CSC vs. SHC mice also showed a shorter distance moved during the OF test (two-way ANOVA; main effect of stress: *F*_1,76_ = 5.011; *P* = 0.028; Bonferroni: *P* = 0.04; Figure 1A).

### Infusion of SHC Donor Feces Slightly Ameliorates CSC-Induced Physiological Effects

**FIGURE 2:**
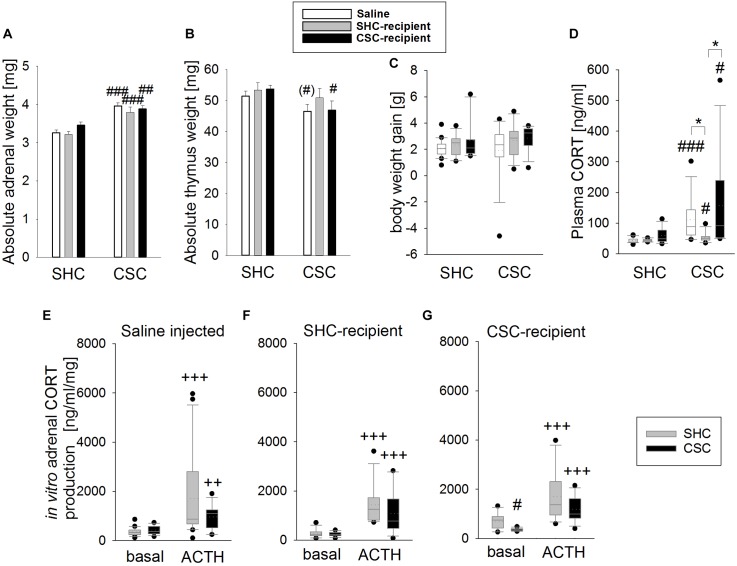
Effects of fecal transplantation (FT) on chronic subordinate colony (CSC) housing-induced physiological alterations. Independent of FT, CSC resulted in an increased absolute adrenal weight **(A)** in all CSC vs. single-housed control (SHC) groups, while infusion of SHC donor feces was able to prevent CSC-induced thymus atrophy **(B)**. Body weight gain during CSC **(C)** was not affected by CSC and/or FT. Basal plasma corticosterone (CORT; **D**) levels were increased in all CSC vs. SHC groups, although this effect was less pronounced in SHC-recipient CSC vs. SHC mice. Moreover, all groups of CSC and SHC mice secreted comparable CORT concentrations in response to *in vitro* adrenocorticotropic hormone (ACTH; 100 nM) stimulation **(E–G)**. SHC (*n* = 11–20); CSC (*n* = 10–13). Parametric data are presented as mean + SEM. Non-parametric data are represented as box-plots. Solid line represents the median, dotted line represents the mean for each data set. Lower boxes indicate 25th, upper boxes indicate 75th percentile, 10th (lower error bar), and 90th percentile (upper error bar) as well as possible outliers beyond the percentiles (indicated by closed circles) are also shown. ^∗^*P* ≤ 0.05 versus, respectively, indicated group; ^(#)^*P* = strong trend, ^#^*P* ≤ 0.05, ^##^*P* ≤ 0.01, ^###^*P* ≤ 0.001 versus respective SHC; ^++^*P* ≤ 0.01, ^+++^*P* ≤ 0.001 versus respective basal.

Chronic subordinate colony housing increased the absolute adrenal weight in all groups compared to the respective SHC group (two-way ANOVA; main effect for stress: *F*_1,69_ = 56.139; *P* < 0.001; Bonferroni: saline-infused: *P* < 0.001; SHC-infused: *P* < 0.001; CSC-infused: *P* = 0.003; Figure 2A), indicating that CSC-induced changes in adrenal weight were independent of the microbiome. In contrast, while CSC-infused CSC (Bonferroni: *P* = 0.05) and saline-infused CSC (by trend: independent *T*-test: *P* = 0.082) mice displayed thymus atrophy when compared to respective SHC mice, SHC-infused CSC mice did not (two-way ANOVA; main effect for stress: *F*_1,73_ = 6.283; *P* = 0.014; Figure 2B). Body weight gain during CSC was neither affected by the factor stress, nor factor FT in any of the experimental groups (Figure 2C). CSC further increased plasma morning CORT in all CSC groups (MWU-test; saline-infused: *P* < 0.001, SHC-infused: *P* = 0.033; CSC-infused: *P* = 0.014) compared to respective SHC groups (Figure 2D), with this effect being less pronounced in SHC-infused CSC mice [KWH-test; H(2): 10.158; *P* = 0.006] compared with saline- (Bonferroni: *P* = 0.022) and CSC-infused CSC mice (Bonferroni: *P* = 0.013). Finally, all SHC (saline-infused: *P* < 0.001; SHC-recipient: *P* < 0.001, CSC-recipient: *P* = 0.001) and CSC (saline-infused: *P* = 0.008; SHC-recipient: *P* < 0.001, CSC-recipient: *P* < 0.001) groups produced higher amounts of CORT following ACTH stimulation (Figures 2E–G), when compared to their respective basal values. CSC-infused CSC vs. respective SHC mice produced lower amounts of CORT under basal conditions (MWU: *P* = 0.013).

### CSC and FT Did Not Affect the Local Inflammatory Status of the Colon

**FIGURE 3:**
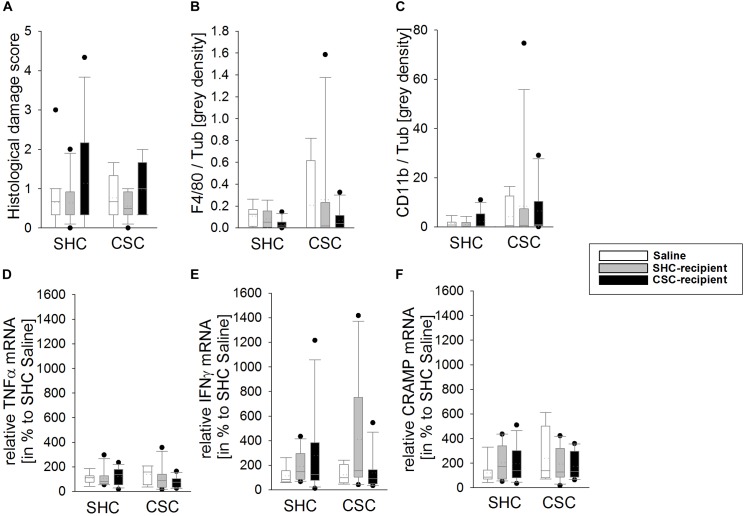
Effects of fecal transplantation (FT) on local colonic inflammatory parameters. The inflammatory status locally in the colon of single-housed control (SHC) and chronic subordinate colony (CSC) housing mice was assessed at the histological, protein and mRNA level. There was no effect of CSC and/or FT on the histological damage score **(A)**, the colonic protein levels of F4/80 **(B)** and cluster of differentiation molecule 11b (CD11b; **C**) as well as the mRNA levels of tumor necrosis factor-α (TNFα; **D**), interferon-γ (IFNγ; **E**) and cathelin-related antimicrobial peptide (CRAMP; **F**) in colonic tissue. SHC (*n* = 7–19); CSC (*n* = 4–13). Parametric data are presented as mean + SEM. Non-parametric data are represented as box-plots. Solid line represents the median, dotted line represents the mean for each data set. Lower boxes indicate 25th, upper boxes indicate 75th percentile, 10th (lower error bar), and 90th percentile (upper error bar) as well as possible outliers beyond the percentiles (indicated by closed circles) are also shown.

Statistical analysis revealed no effect of the factors stress and/or FT on the histological damage score (Figure 3A), the colonic protein levels of F4/80 (Figure 3B) and CD11b (Figure 3C) as well as the mRNA levels of TNFα (Figure 3D), IFNγ (Figure 3E), and CRAMP (Figure 3F) in colonic tissue.

### Infusion of SHC and CSC Donor Feces Affected CSC-Induced Systemic Inflammation in the Plasma, Respectively

**FIGURE 4:**
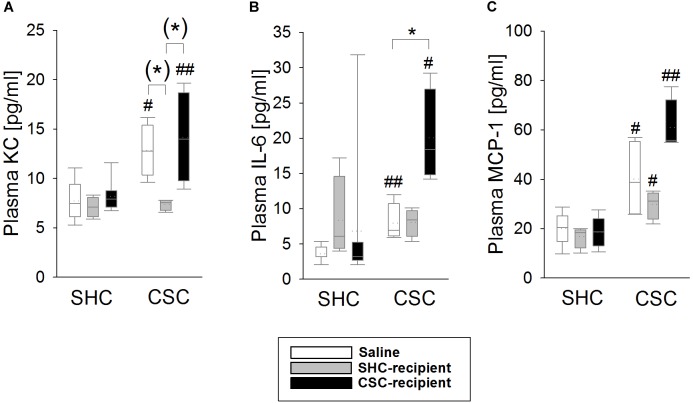
Effects of fecal transplantation (FT) on chronic subordinate colony (CSC) housing-induced systemic inflammation. Systemic inflammatory markers were measured in the plasma of single-housed control (SHC) and CSC mice. Plasma levels of keratinocyte chemoattractant (KC; **A**), interleukin-6 (IL-6; **B**) as well as monocyte chemotactic protein-1 (MCP-1; **C**) levels were increased in saline-infused CSC compared with respective SHC and CSC-infused CSC compared with respective SHC animals. In contrast, SHC-infused CSC compared with respective SHC animals only showed increased MCP-1, but not KC and IL-6 levels, and had lower KC levels than saline- and CSC-infused CSC mice. SHC (*n* = 4–8); CSC (*n* = 4). Parametric data are presented as mean + SEM. Non-parametric data are represented as box-plots. Solid line represents the median, dotted line represents the mean for each data set. Lower boxes indicate 25th, upper boxes indicate 75th percentile, 10th (lower error bar), and 90th percentile (upper error bar) as well as possible outliers beyond the percentiles (indicated by closed circles) are also shown. ^(∗)^*P* = strong trend, ^∗^*P* ≤ 0.05 versus, respectively, indicated group; ^#^*P* ≤ 0.05, ^##^*P* ≤ 0.01 versus respective SHC.

Statistical analysis revealed that KC, IL-6 as well as MCP-1 levels were increased in the plasma of saline-infused CSC [MWU test: KC: *P* = 0.016 (Figure 4A); IL-6: *P* = 0.004 (Figure 4B); MCP-1: *P* = 0.028 (Figure 4C)] and CSC-recipient CSC (MWU test: KC: *P* = 0.008; IL-6: *P* = 0.048; MCP-1: *P* = 0.004) vs. respective SHC animals. SHC-recipient CSC animals only showed increased MCP-1 (MWU: *P* = 0.029) but not KC and IL-6 levels when compared to their respective controls. Statistical analysis using KWH test further revealed a significant main effect of FT on plasma KC [H(2): 7.411; *P* = 0.025]; as well as plasma IL-6 levels [H(2): 7.449; *P* = 0.024] in CSC animals. Thereby, saline-infused CSC (Bonferroni: *P* = 0.055) as well as CSC-recipient CSC animals (Bonferroni: *P* = 0.055) had higher plasma KC levels than SHC-recipient CSC animals. Moreover, CSC-recipient CSC animals had significantly higher IL-6 levels than saline-infused CSC animals (Bonferroni: *P* = 0.042) and, by trend, higher IL-6 levels than SHC-recipient CSC animals (Bonferroni: *P* = 0.072).

### Infusion of SHC Donor Feces Ameliorated CSC-Induced Effects on Bone Homeostasis. In Turn, Infusion of CSC Donor Feces Induced Stress Effects on Bone Homeostasis in Unstressed SHC Mice

**FIGURE 5:**
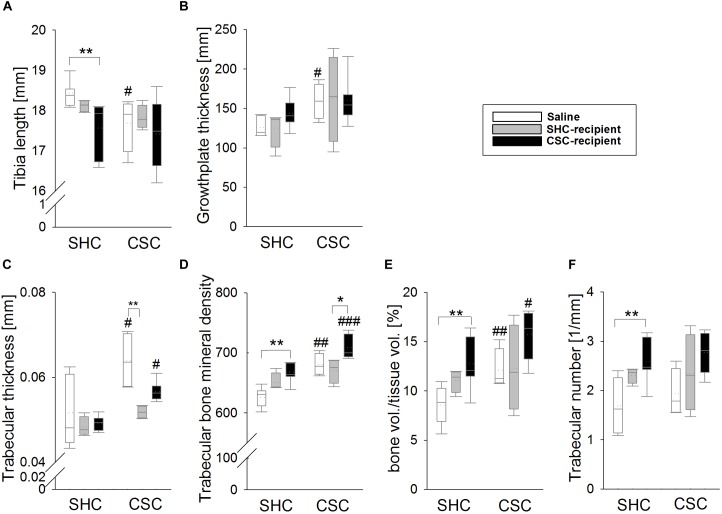
Effects of fecal transplantation (FT) on chronic subordinate colony (CSC) housing-induced changes in the bone. Saline-infused CSC compared with respective single-housed control (SHC) mice had significantly shorter tibias **(A)** and an increased femoral growth-plate width **(B)**. Trabecular thickness **(C)**, trabecular bone mineral density (BMD) **(D)** as well as trabecular bone volume/tissue volume (BV/TV; **E**) were significantly increased in both saline-infused CSC compared with respective SHC mice as well as in CSC-infused CSC compared with respective SHC animals, but not in SHC-infused CSC versus SHC mice. Furthermore, the infusion of CSC feces compared with saline aggravated trabecular BMD, the trabecular BV/TV and the trabecular number **(F)** in SHC animals. SHC (*n* = 4–8); CSC (*n* = 4–8). Parametric data are presented as mean + SEM. Non-parametric data are represented as box-plots. Solid line represents the median, dotted line represents the mean for each data set. Lower boxes indicate 25th, upper boxes indicate 75th percentile, 10th (lower error bar), and 90th percentile (upper error bar) as well as possible outliers beyond the percentiles (indicated by closed circles) are also shown. ^∗^*P* ≤ 0.05, ^∗∗^*P* ≤ 0.01 versus respectively indicated group; ^#^*P* ≤ 0.05, ^##^*P* ≤ 0.01, ^###^*P* ≤ 0.001 versus respective SHC.

Saline-infused CSC compared to SHC animals had significantly smaller tibia length (MWU: *P* = 0.024; Figure 5A) and an increased femoral growth-plate width (MWU: *P* = 0.038; Figure 5B). This effect was not visible in SHC-recipient and CSC-recipient CSC compared to SHC animals, respectively. Moreover, CSC-recipient SHC animals displayed significantly smaller tibia length compared to saline-infused SHC animals [KWH-test (H(2): 9.882; *P* = 0.007; Bonferroni: *P* = 0.005]. Statistical analysis further revealed, that trabecular thickness (Figure 5C), trabecular BMD (Figure 5D) as well as trabecular bone volume/tissue volume (BV/TV, Figure 5E) were significantly increased in both CSC vs. SHC saline-infused (MWU: Trabecular thickness: *P* = 0.048; BMD: *P* = 0.004; BV/TV: *P* = 0.008) as well as CSC-recipient animals (MWU: Trabecular thickness: *P* = 0.014; BMD: *P* < 0.001; BV/TV: P = 0.054). However, these effects were absent in SHC-recipient CSC vs. SHC animals. SHC-recipient CSC animals moreover displayed a significantly smaller trabecular thickness compared to saline-infused CSC animals [KWH-test: H(2): 9.723; *P* = 0.008; Bonferroni: *P* = 0.006] and a smaller trabecular BMD compared to CSC-recipient CSC animals [KWH-test: H(2): 8.406; *P* = 0.015; Bonferroni: *P* = 0.023]. Furthermore, CSC-recipient vs. saline-infused SHC animals displayed an increased trabecular BMD [KWH-test: H(2): 11.836; *P* = 0.003; Bonferroni: *P* = 0.002], trabecular BV/TV [KWH-test: H(2): 10.023; *P* = 0.007; Bonferroni: *P* = 0.006] and trabecular number [KWH-test: H(2): 10.533; *P* = 0.005; Bonferroni: *P* = 0.004; Figure 5F].

## Discussion

In the present study, we showed that repeated FT from SHC donors into CSC-recipient mice has mild stress protective effects, ameliorating CSC-induced thymus atrophy, anxiety-related behavior, systemic low-grade inflammation as well as alterations in bone homeostasis. In turn, FT from CSC donors induced a “stressed” bone phenotype in SHC-recipient mice and slightly aggravated CSC-induced systemic low-grade inflammation and changes in bone homeostasis in CSC-recipient mice. In contrast to previous CSC studies (Reber et al., 2007; Uschold-Schmidt et al., 2012), basal plasma CORT concentrations were increased compared to respective SHC mice, and ACTH-stimulated *in vitro* CORT production was comparable between CSC mice of all groups, suggesting that the procedure of rectal infusion *per se* interferes with CSC-induced HPA axis activity.

Numerous studies showed that the gut microbiome plays an important role in the intestinal barrier homeostasis (Backhed et al., 2004; Bercik et al., 2012), the maturation and functionality of the immune system (Round and Mazmanian, 2009; Round et al., 2010; Olszak et al., 2012), bone homeostasis (Sjogren et al., 2012; Li et al., 2016; Novince et al., 2017), acute stress responsiveness (Sudo et al., 2004), neurogenesis (Ogbonnaya et al., 2015) as well as in the regulation of the host’s behavior and mood (Cryan and Dinan, 2012; Stilling et al., 2014). Consequently, it is not surprising, that the transplantation of stool is able to affect the recipient’s physiology (Sudo et al., 2004), immunology (Tian et al., 2016; De Palma et al., 2017) and behavioral characteristics like exploratory (Bercik et al., 2011; Collins et al., 2013) or depressive-like behavior (Kelly et al., 2016). Given the potential of CSC to alter the overall composition of the intestinal microbiome (Reber et al., 2016b), and, amongst others, to affect anxiety-related behavior (Langgartner et al., 2015), the first aim of the present study was to investigate if the anxiogenic effects of CSC can be prevented and/or transmitted to non-stressed mice by the transplantation of SHC and CSC feces, respectively. To do so, we analyzed the animal’s anxiety-related behavior in the OF/NO test on day 19. In line with previous publications (Langgartner et al., 2015, 2017), CSC increased the anxiety-related behavior in saline-treated animals, ensuring that the procedure of rectal infusion *per se* did not have an effect in this context. In detail, saline-treated CSC versus SHC animals displayed an increased time in the corners of the arena and a decreased number of entries into the inner zone during OF testing as well as a decreased locomotion and novel object exploration and an increased time in the corners during the subsequent 5 min of NO exploration. Importantly, the total distance moved during the OF test did not differ between saline-infused CSC and SHC animals, indicating that the outcome of the above mentioned anxiety-related parameters was not affected by differences in general locomotion (Langgartner et al., 2017). Interestingly, although infusion of CSC donor feces into both SHC and CSC recipients did not aggravate basal or CSC-induced anxiety-related behavior, infusion of SHC donor feces at least slightly ameliorated CSC-induced anxiety. More specific, infusion of SHC feces prevented the CSC-induced decrease in the numbers of entries into the inner zone and NO exploration during the OF and NO test, respectively. However, as SHC-infused CSC versus SHC mice displayed a decreased distance moved in the OF test, we at this point cannot exclude that the protective effects of SHC stool infusion on CSC-induced anxiety are affected by a general decrease in locomotion in these mice.

Adrenal hypertrophy and thymus atrophy are typical indicators of chronic stress in general (Selye, 1976; Engler and Stefanski, 2003; Stefanski et al., 2003; Ulrich-Lai et al., 2006), and of CSC in particular (Reber et al., 2007; Langgartner et al., 2015, 2017). In confirmation that the CSC model worked reliably in the present study, saline-infused CSC compared to SHC mice displayed an increased adrenal and decreased thymus weight. In line with the above reported anxiety data, infusion of CSC feces did not affect these parameters in SHC nor in CSC mice. However, in line with the hypothesis that infusion of SHC stool has stress-protective properties, SHC-infused CSC animals developed adrenal hypertrophy, but no thymus atrophy. In support of the latter, colonization of GF mice with conventional microbiota is able to influence the size of the thymus in a gender-specific manner (Fransen et al., 2017). Of note, body weight gain was neither affected by FT nor CSC, which is, at least for the latter, in line with previous studies indicating that a reduced body weight during CSC is not a very reliable parameter (Slattery et al., 2012; Langgartner et al., 2015).

Nineteen days of CSC have repeatedly shown to result in unaltered basal morning plasma CORT levels (Reber et al., 2007; Langgartner et al., 2017). All three groups of CSC mice in the current study, however, displayed increased basal CORT concentrations compared to their respective SHC groups. Of note in this context, videotaping the CSC colony in the hour before euthanasia allows us to exclude that the elevated CORT levels in the present study are due to an acute attack by the resident shortly before killing. Given that not only SHC- and CSC-, but also saline-infused CSC mice showed this effect, we assume that the combination of rectal infusions and CSC exposure affected the HPA-axis in a different way than CSC exposure alone. In support, all groups of CSC mice did also not develop a decreased adrenal *in vitro* ACTH responsiveness (Uschold-Schmidt et al., 2012). Thus, although a recent study by Sudo et al. (2004) revealed that FT at an early developmental stage is able to alter the physiological stress-responsiveness toward acute stressors in mice, FT during adulthood seems to have little impact on HPA axis (re)activity, both during basal conditions and following chronic psychosocial stress.

Chronic psychosocial stress in humans is characterized by systemic immune activation and chronic low-grade inflammation (Bellingrath et al., 2013; Rohleder, 2014). Of note in this context, chronic psychosocial stress in male mice induced by the social disruption stress (SDR) paradigm, affects the gut microbial composition and, as a consequence, increased levels of circulating pro-inflammatory IL-6 and MCP-1 (Bailey et al., 2011). As 19 days of CSC also result in systemic low-grade immune activation, characterized by elevated concentrations of different pro- and anti-inflammatory mediators in the plasma (Langgartner et al., 2018) and changes in the gut microbial composition (Reber et al., 2016b), we hypothesized that these two CSC consequences are also causally linked with changes in the microbiome driving low-grade systemic inflammation. Confirming that the rectal infusion itself had no influence in this context, CSC reliably increased plasma levels of KC, IL-6 and MCP-1 in saline-infused mice. Importantly, and in support of the hypothesis that CSC-induced changes in the gut microbiome indeed are critically involved in promoting systemic inflammation, infusion with SHC feces prevented CSC-induced elevation of plasma KC and IL-6. Moreover, although in turn infusion of CSC donor feces did not induce a “stressed” cytokine profile in SHC mice, it aggravated the CSC-induced elevation of plasma IL-6.

In line with a recent study (Langgartner et al., 2017), CSC under SPF conditions in the present study did not cause any signs of local colonic inflammation, a finding that was not affected by the rectal infusion procedure *per se*. In detail, saline-infused CSC versus SHC mice did not differ in the histological damage score of the colon, the colonic mRNA expression of TNFα and IFNγ, as well as the colonic intestinal barrier function, indexed by colonic CRAMP mRNA expression. Moreover, colonic F4/80 and CD11b protein expression were also comparable between saline-infused CSC and SHC mice, suggesting no differences in the colonic macrophage and monocyte infiltration between the groups. In line with this lack of CSC-induced colitis, infusion of SHC or CSC mice with SHC or CSC feces did not result in any signs of colonic inflammation. Besides being a risk factor for mental and inflammatory disorders, chronic stress or chronic stress-associated mental disorder as PTSD (Glaesmer et al., 2011, 2012; Calarge et al., 2014; Gebara et al., 2014; Zong et al., 2016) are linked to bone disorders (Batty et al., 2009; Azuma et al., 2015) and arthritis in humans. In line with this, our group was recently able to show that CSC negatively influences endochondral ossification (Foertsch et al., 2017), indicated by an increased appositional and decreased longitudinal bone growth. Confirming that the rectal infusion *per se* did not affect CSC effects on bone homeostasis, saline-infused CSC compared with respective SHC mice showed a decreased tibia length as well as an increased growth plate width, trabecular thickness, BMD and bone volume to tissue volume, similarly as described in our previous study (Foertsch et al., 2017). In support of the hypothesis that CSC-induced bone effects are mediated by the gut microbiome, infusion of SHC feces was able to prevent CSC effects on bone homeostasis. Moreover, infusion of CSC feces not only aggravated CSC-induced effects on trabecular BMD, it also induced a “stressed” bone phenotype in SHC-recipient mice. The latter was indicated by an increased BMD, BV/TV and trabecular number as well as a reduced tibia length in CSC-infused SHC mice compared with respective saline-infused SHC mice. Indeed, it was shown previously that the gut microbiome in general significantly effects on bone homeostasis and that alterations in the gut microbial community influence the latter. In detail, germ-free vs. conventionally raised mice exhibit increased bone mass, whereas re-colonization normalized bone parameters (Sjogren et al., 2012). Moreover, estrogen-depletion does not result in bone loss in germ-free mice and supplementation with probiotics prevents from ovariectomy-induced bone loss (Ohlsson et al., 2014; Li et al., 2016). Interestingly, changes in pro-inflammatory and pro-osteoclastic cytokines like TNFα, IL-6, and IL-1β were proposed as one underlying molecular mechanism (Ohlsson et al., 2014; Li et al., 2016; Xiao et al., 2017). Thus, the observed effects of FT on inflammatory cytokines in the present study might also, at least partly, account for the effects on bone homeostasis.

However, despite these promising results, there are still some limitations that have to be addressed in the future. In contrast to other human (Keshteli et al., 2017) and animal (Kelly et al., 2016) studies employing FT, we in the present study abstained from treating recipient animals with antibiotics prior to FT. Nevertheless, although antibiotic treatment decreases the overall gut microbial richness and diversity (Francino, 2015) and thus, potentially opens niches for infused germs to re-colonize the recipient’s intestinal tract, it was recently shown in rodents, that the prior administration of antibiotics affects the establishment of transplanted donor phylotypes only in rather minor ways (Manichanh et al., 2010). However, it still remains unclear if this is also the case in our experimental setup. Another limitation is that we employed rectal instead of oral FT. The latter is more common in rodent studies (Bercik et al., 2011; Allen et al., 2012; Collins et al., 2013; Kelly et al., 2016; Galley et al., 2017) and also easily practicable in humans and thus of high translational relevance. Finally, it needs to be addressed if the FT method used in the present study is able to change the microbial composition of the host, and if so, what bacterial genera and species are involved in the protective/aggravating effects found in the present study.

## Conclusion

In conclusion, although future studies are needed to optimize the stress-protective effects of FT, our findings revealed that infusion of “unstressed” donor feces might be a promising tool to prevent/treat the adverse outcomes of chronic psychosocial stress on systemic low-grade inflammation as well as bone homeostasis. Moreover, given that some negative consequences of chronic psychosocial stress could be transferred via FT, we propose that clinical approaches employing FT as a therapeutic tool, i.e., to treat recurrent *Clostridium difficile*-induced colitis (Dodin and Katz, 2014), should carefully screen fecal donor participants for their possible stress/trauma history. Thus, our findings (summarized in Table 1) on the one hand significantly contribute to understand the interplay between the gut microbiome and the host during chronic stress exposure and on the other hand expand the possible field of FT application.

**Table 1:** Summary of findings supporting the role of the intestinal microbiome as mediator of chronic psychosocial stress-induced pathology.

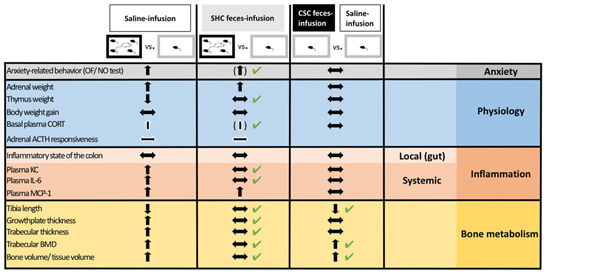

*Table 1 summarizes the main effects of single housed control (SHC)- and chronic subordinate colony housing (CSC)-feces-infusions on various behavioral, physiological, inflammatory and bone metabolic parameters in SHC and CSC mice. Black arrows indicate effect directions. White frames surrounding certain arrows in the left and middle columns indicate that CSC effects observed in “infused” mice in the present study are different from previously published CSC effects in “non-infused” mice. The left column compares saline-treated CSC animals with respective SHC mice. The middle column compares SHC feces-infused CSC with respective SHC mice. The right column compares CSC feces-infused SHC with saline-infused SHC mice. Green checkmarks in the middle and right column indicate findings supporting a causal role of the intestinal microbiome as mediator of CSC-induced pathology. Arrows in brackets represent partial but not full prevention/recovery.*

## Author Contributions

DL and SR planned the study. DL, CV, A-LW, JK, SF, SB, and MH-L performed the experiments. DL and CV did the statistical analysis. DL, SF, MH-L, AI, and SR wrote the manuscript.

## Conflict of Interest Statement

The authors declare that the research was conducted in the absence of any commercial or financial relationships that could be construed as a potential conflict of interest.
